# Aid Effectiveness in the Sustainable Development Goals Era: Comment on ""It’s About the Idea Hitting the Bull’s Eye": How Aid Effectiveness Can Catalyse the Scale-up of Health Innovations"

**DOI:** 10.15171/ijhpm.2018.130

**Published:** 2018-12-25

**Authors:** Osondu Ogbuoji, Gavin Yamey

**Affiliations:** Center for Policy Impact in Global Health, Duke Global Health Institute, Durham, NC, USA.

**Keywords:** Aid Effectiveness, Sustainable Development Goals, Development Assistance for Health, Health Financing, Global Health

## Abstract

Over just a six-year period from 2005-2011, five aid effectiveness initiatives were launched: the Paris Declaration on Aid Effectiveness (2005), the International Health Partnership plus (2007), the Accra Agenda for Action (2008), the Busan Partnership for Effective Cooperation (2011), and the Global Partnership for Effective Development Cooperation (GPEDC) (2011). More recently, in 2015, the Addis Ababa Action Agenda (AAAA) was signed at the third international conference on financing for development and the Universal Health Coverage (UHC) 2030 Global Compact was signed in 2017. Both documents espouse principles of aid effectiveness and would most likely guide financing decisions in the Sustainable Development Goals (SDG) era. This is therefore a good moment to assess whether the aid effectiveness agenda made a difference in development and its relevance in the SDG era.

In their three-country qualitative study published in August 2018 in this journal, Wickremasinghe and colleagues explored whether there were any linkages between six aid effectiveness principles and the factors that promote or inhibit the scale-up of maternal and newborn health (MNH) interventions.^1^ The six principles of aid effectiveness that the authors examined were country ownership over programs; alignment between donor funding and country priorities; harmonization of donor activities (to avoid duplication and fragmentation of efforts); transparency and accountability; providing predictable, long-term funding; and engaging civil society.

This is one of very few studies that have looked at this issue from the recipient country’s perspective – a perspective that is often lacking in discussions about funding. Not surprisingly, their study showed that when the government, donors, implementers, and civil society adopt aid effectiveness principles, the probability of scaling up externally introduced innovations significantly increases. Their results thus help to reaffirm the value of paying attention to the key principles contained in the aid effectiveness agenda.

## The Aid Effectiveness Agenda Has Stalled

Over just a six-year period from 2005-2011, five aid effectiveness initiatives were launched: the Paris Declaration on Aid Eﬀectiveness (2005),^2^ the International Health Partnership plus (2007),^3^ the Accra Agenda for Action (2008),^2^ the Busan Partnership for Eﬀective Cooperation (2011),^4^ and the Global Partnership for Eﬀective Development Cooperation (GPEDC) (2011).^5^ However, after this flurry of activity, the momentum around the aid effectiveness agenda stalled and it was unclear where it was heading. As Neils Keijzer of the German Development Institute and Erik Lundsgarde of the Danish Institute for International Studies pointed out in their analysis of the GPEDC, “Efforts to promote development effectiveness have stagnated as donor peer pressure eroded and developing country leadership waned.”^6^ They argued that the GPEDC had difficulty gaining traction, because of the uneven engagement of stakeholders, and that there was a critical need to “revive the development effectiveness agenda and broaden the coalition interested in the ‘how’ of development cooperation.”

More recently, in 2015, the Addis Ababa Action Agenda (AAAA) was signed at the third international conference on financing for development.^7^ This agenda, which supercedes the previous Doha Declaration of 2008,^8^ and the Monterrey Consensus of 2002,^9^ is the major framework guiding financing for development in the post-2015 Sustainable Development Goal (SDG) era. In the AAAA, development partners and country governments restated their commitment to pursuing aid effectiveness principles. In a related push within the health sector, a recognition of the importance of the SDG target for universal health coverage (UHC) led to the signing of the UHC 2030 Global Compact in 2017. In this compact as well, principles similar to those contained in the Paris Declaration of Aid Effectiveness, such as transparency, accountability, and cooperation, were espoused. It is therefore necessary to assess whether the aid effectiveness agenda has made a difference in development.

It is currently unclear from the available evidence whether aid effectiveness principles have improved the efficiency and impact of aid. Overall, the evidence gives a mixed picture suggesting partial success at best, particularly in terms of promoting transparency.^10,11^ A formal evaluation of the implementation of the Paris Aid Effectiveness Agenda across 22 countries and 18 donors found marked variations in the levels of implementation and the quality of results obtained by relevant stakeholders.^12^ There is evidence that donor harmonization has not improved and that donor alignment with country priorities may actually have deteriorated. For example, Martinez-Alvarez and colleagues studied donor funding for reproductive, maternal, newborn and child health (RMNCH) over the period 2008-2013, a period that saw a substantial rise in aid for RMNCH.^13^ They studied (*a*) the degree of alignment between aid and the recipient country’s public health financial management systems, and (*b*) the degree of donor harmonization. Their results showed that from 2008-2013, country alignment worsened (as measured by the proportion of donor financing channeled through governments) while donor harmonization was generally low overall and remained low during the study period.

## The Development Landscape Is Rapidly Changing

Given the weakness of the evidence, it is therefore encouraging that key informants interviewed by Wickremasinghe and colleagues found that adopting aid effectiveness principles at the country level made significant positive contributions to scale-up of MNH health programs. But this study was conducted five to six years ago, during the Millennium Development Goals (MDGs) era, and much has changed in the development landscape since then. The narrow set of eight MDGs has been superseded by an expansive set of 17 SDGs, which have 169 targets. Development assistance for health has plateaued and in some cases is now reducing, with projections of further decreases in the future.^14^ Emerging donors such as China and the Gulf States have become major actors in development finance, which could potentially have a dramatic effect on global health cooperation.^15^ South-South financing of development is occurring more frequently.^16^ In the coming years, over a dozen middle-income countries are expected to transition out of eligibility for donor financing.^17^ These shifts all have implications for the aid effectiveness agenda.

The SDGs are highly ambitious and they emphasize the concept of leaving no one behind. In order to achieve these goals, significantly more resources will be needed. To achieve the health-related SDG alone (SDG 3), Stenberg and colleagues estimate that by 2030, an *additional* $371 billion will be required annually across the 67 low-income countries (LICs) and middle-income countries (MICs) that represent 95% of the total population of all LICs and MICs.^18^ To reach all the SDGs, the resource need will be in the trillions of dollars. There is an expectation that funding from non-traditional donors will become increasingly significant^19^; non-traditional donors (eg, Brazil, China, Saudi Arabia, and the United Arab Emirates) are those who are not members of the Development Assistance Committee of the Organization for Economic Cooperation and Development (the DAC).^19^ Such nontraditional donors have not generally signed up to aid effectiveness principles, there is little indication to suggest they will do so in the future, and they are engaging in new ways of “doing business” when it comes to making grants or loans. It is conceivable that the traditional DAC donors who espoused the current aid effectiveness principles will play a smaller and smaller role in funding SDG programs and thus their leverage will be reduced.

Another important change is the transition of MICs from aid; these countries become ineligible for multilateral donor concessional financing as their gross domestic product per capita reaches a particular threshold, and as they meet other donor-specific criteria. With this transition, countries are expected to assume greater responsibility for domestic health spending through domestic resource mobilization and allocation. This shift will alter the dynamic between donors and host governments—the leverage enjoyed by donors falls in proportion to the reduction in the proportion of public revenue sourced from donors. Indeed, Wickremasinghe and colleagues found that as donors reduced their funding to India and Nigeria, their leverage over these governments diminished.

Taken together, these changes in projected aid flows, need for aid, eligibility for aid, and entry of new donors, all conspire to reduce the leverage that donors typically wield over recipient countries.

## A New Aid Effectiveness Agenda for a New Era of Development Cooperation

Given the changing landscape of development, including projected decreases in aid, the rise of non-traditional donors, the transition of MICs away from aid, and the increase in private financing of development objectives, the “old” aid effectiveness agenda will need to be revised and updated to be more inclusive of these shifts. A revised agenda should also address some of the shortcomings of the previous one, such as the lack of an accountability mechanism and the lack of attention to marginalized populations. Global adoption and implementation of aid effectiveness principles in the MDGs era was mired in politics and certain principles never took hold. The following quotation from a country representative of a donor agency, which comes from a formal evaluation of aid effectiveness, aptly describes the influence of politics: “Certain decisions made by headquarters for political or geostrategic reasons limit agencies’ actions in the field.”^12^ In her recent analysis of which principles “stuck” and which have been sidelined, Annalisa Prizzon of the Overseas Development Institute concluded that the “principles of development that have fallen off the radars of developing country governments” are “harmonisation, results, transparency and untied aid.”^20^ Without a strong accountability mechanism, it will be impossible to ensure compliance with any new aid effectiveness agenda in the SDG era, given the multiple actors, goals, and interests. There will also need to be a new focus on disadvantaged populations in order to align any new aid effectiveness agenda with the SDG movement to “Leave No One Behind.” The UHC 2030 Global Compact explicitly lists this as one of its principles, and AAAA restates the global push towards achieving equality, but we believe that this principle of leaving no one behind should be made more prominent in aid effectiveness discussions in order to promote inclusive development.

Some may argue that a new aid effectiveness agenda may not be required since the fraction of aid as a proportion of total development finance is expected to fall significantly over time. However, Wickremasinghe and colleagues’ study shows that even when donor leverage might be limited because of the reduction of donor funds, that leverage does not disappear entirely. It is possible for donors to still make significant positive contributions through other means such as providing technical capacity. Finally, for MICs facing transition away from development assistance for health, it will be important to reiterate the aid effectiveness principle of providing predictable financing. Therefore, donors should as a matter of principle jointly develop transition plans with their host countries to ensure that progress towards achieving the SDGs are not stalled or reversed as a result of donor exits. Overall, we therefore believe that it is worthwhile to revisit the aid effectiveness agenda, in partnership with all the key players in the space, to rearticulate an agenda better suited to the SDGs era.

## Ethical issues

Not applicable.

## Competing interests

The Center for Policy Impact in Global Health has received grant funding from multiple aid agencies: the Bill & Melinda Gates Foundation; Gavi, the Vaccine Alliance; and the World Health Organization (WHO). OO has also been funded by The Global Fund to Fight AIDS Tuberculosis and Malaria, and Save the Children International.

## Authors’ contributions

OO and GY developed the initial concept. OO prepared the first draft. Both authors contributed to subsequent drafts and approve of the final draft.
